# Clinicopathologic features and prognostic value of claudin 18.2 overexpression in patients with resectable gastric cancer

**DOI:** 10.1038/s41598-023-47178-6

**Published:** 2023-11-16

**Authors:** Hyung-Don Kim, Eugene Choi, Jinho Shin, In-Seob Lee, Chang Seok Ko, Min-Hee Ryu, Young Soo Park

**Affiliations:** 1 Department of Oncology, Asan Medical Center, University of Ulsan College of Medicine, 88, Olympic-Ro 43-gil, Songpa-gu, Seoul, 05505 Republic of Korea; 2 Department of Pathology, Asan Medical Center, University of Ulsan College of Medicine, 88, Olympic-Ro 43-gil, Songpa-gu, Seoul, 05505 Republic of Korea; 3 Department of Surgery, Asan Medical Center, University of Ulsan College of Medicine, 88, Olympic-Ro 43-gil, Songpa-gu, Seoul, 05505 Republic of Korea

**Keywords:** Gastric cancer, Cancer therapy, Prognostic markers, Tumour biomarkers

## Abstract

Claudin 18.2 has emerged as a promising therapeutic target in gastric cancer based on phase 3 studies. However, clinicopathologic features associated with claudin 18.2 overexpression have not been comprehensively studied specifically for patients with resectable gastric cancer. This retrospective study included 299 patients with stage I–III resectable gastric cancer who underwent curative surgical resection. Possible associations between claudin 18.2 overexpression (moderate-to-strong expression in ≥ 75% by the 43-14A clone) and clinicopathologic features and survival outcomes were analyzed. There were 90 (30.1%), 96 (32.1%), and 113 (37.8%) patients with stage I, II, and III disease, respectively. Claudin 18.2 overexpression was noted in 139 out of 299 patients (46.5%). Claudin 18.2 overexpression was associated with a younger age, a lower invasion depth limited to the mucosa/submucosa, and less frequent lymphovascular invasion. Claudin 18.2 overexpression was also associated with Borrmann type 4 among patients with advanced gastric cancer and the diffuse histological type. Claudin 18.2 overexpression was not an independent factor for survival outcomes. In conclusion, claudin 18.2 was overexpressed in almost half of resectable gastric cancer patients. Claudin 18.2 overexpression was associated with some clinicopathological characteristics, but was not an independent prognostic factor in a localized setting.

## Introduction

Gastric cancer is the 5th most common cancer and the 4th leading cause of cancer-related death worldwide according to research conducted in 2020^1^. Over the past decade, there has been significant progress in systemic chemotherapy for patients with advanced gastric cancer, especially with targeted and immunotherapeutic agents. For human epidermal growth factor 2 (HER2)-positive tumors, a survival benefit of adding trastuzumab to chemotherapy for patients with HER2-positive tumors was demonstrated in the first-line setting^2^, and trastuzumab–deruxtecan also showed a survival benefit over the physician’s choice of chemotherapy in the 3rd line setting^3^. Recently, in the CheckMate-649 study, the addition of nivolumab to chemotherapy prolonged overall survival (OS) in advanced gastric cancer patients in the front-line setting^4^. These findings have increased enthusiasm for applying novel agents to patients with advanced gastric cancer.

Claudin 18.2 is a tight junction protein that regulates tissue permeability, paracellular transport, and signal transduction. Claudin 18.2 is selectively expressed at tight junctions in normal gastric epithelium, but it frequently becomes exposed and overexpressed on the surface of gastric adenocarcinoma cells along with the disruption of tight junctions during the process of malignant transformation^5^. Zolbetuximab is a monoclonal antibody that targets claudin 18.2 and has anti-tumor activity by inducing antibody-dependent cellular cytotoxicity and complement-dependent cytotoxicity^6^. Recently, two phase 3 studies (SPOTLIGHT^7^ and GLOW^8^) demonstrated that the addition of zolbetuximab to chemotherapy prolonged the OS of patients with unresectable or metastatic gastric and gastroesophageal junction cancer in the first-line setting.

Given the potent efficacy of adding zolbetuximab to chemotherapy, the expansion of its indications to earlier stages of disease should be considered for patients with localized resectable gastric cancer. However, this would require the delineation of the clinicopathological characteristics of claudin 18.2-positive tumors in a curative setting. Recently, Kubota et al. characterized the features of claudin 18.2-positive tumors in a palliative setting based on a method used in the phase 3 studies of zolbetuximab (moderate-to-strong expression of claudin 18.2 in ≥ 75% of tumor cells), revealing that claudin 18.2 overexpression is associated with Borrmann type 4 gross type and *KRAS* amplification without a prognostic impact on survival outcomes^9^. However, considering the substantial differences in the tumor biology and clinical course between localized and metastatic tumors, the clinicopathologic features of claudin 18.2-positive gastric cancers need to be characterized in the resectable setting before claudin 18.2-directed treatment can be considered in a localized setting. Although previous studies have evaluated the features of claudin 18.2 expression in a localized setting, the clinical implications of those findings are limited by the use of different antibody clones or different cut-off points for claudin 18.2 positivity^10–14^ or by reporting a mixed analysis of localized and metastatic settings^9,15^, precluding its detailed characterization specific to the localized setting.

In this study, we aimed to investigate the rate of claudin 18.2-positivity and the clinicopathologic features and survival outcomes of claudin 18.2-positive localized resectable gastric cancer using the same methods as in the recent phase 3 studies^7,16^.

## Results

### Clinical characteristics of the study patients

In the overall study population, the median age was 63 years and 68.6% were male (Table 1). About three-fourths of patients had advanced gastric cancer (AGC), and Bormann type 3 was the most frequent AGC subtype (47.2%), followed by Boramann type 2 (21.4%) and Bormann type 4 (14.8%). Lower/middle location was the most frequent location (79.3%) of the primary tumor. Diffuse type was the most frequent Lauren classification subtype (40.1%). There were 90 (30.1%), 96 (32.1%), and 113 (37.8%) patients with stage I, II, and III disease, respectively. Following surgical resection, 198 patients (66.2%) received adjuvant chemotherapy. Among them, 118 patients (59.6%) were treated with adjuvant S-1 and 80 (40.4%) with capecitabine plus oxaliplatin.

**Table 1:** Clinicopathologic characteristics according to claudin 18.2 positivity.

Characteristics	Overall study population (n = 299)	Claudin 18.2 negative (n = 160)	Claudin 18.2 positive (n = 139)	P-value
Median age (years)	63 (27–95)	66 (33–95)	61 (27–83)	< 0.001
Male sex	205 (68.6)	118 (73.8)	87 (62.6)	0.051
Gross type				0.014
EGC	70 (23.4)	28 (17.5)	42 (30.2)	
AGC	229 (76.6)	132 (82.5)	97 (69.8)	
AGC subtype	(n = 229)	(n = 132)	(n = 97)	0.008
Borrmann type 1	9 (3.9)	8 (6.1)	1 (1.0)	
Borrmann type 2	49 (21.4)	36 (27.3)	13 (13.4)	
Borrmann type 3	108 (47.2)	57 (43.2)	51 (52.6)	
Borrmann type 4	34 (14.8)	14 (10.6)	20 (20.6)	
EGC-like or unclassifiable	29 (12.7)	17 (12.9)	12 (12.4)	
Location				0.537
Lower/middle	237 (79.3)	129 (80.6)	108 (77.7)	
Upper	58 (19.4)	30 (18.8)	28 (20.1)	
Entire	4 (1.3)	1 (0.6)	3 (2.2)	
WHO classification				0.089
WD/MD/papillary	110 (36.8)	68 (42.5)	42 (30.2)	
PD/PD with SRC/SRCa	166 (55.5)	81 (50.6)	85 (61.2)	
Others	23 (7.7)	11 (6.9)	12 (8.6)	
Lauren classification				0.011
Intestinal type	113 (37.8)	72 (45.0)	41 (29.5)	
Diffuse type	120 (40.1)	53 (33.1)	67 (48.2)	
Mixed/indeterminate type	66 (22.1)	35 (21.9)	31 (22.3)	
Invasion depth				0.035
Mucosa/submucosa	70 (23.4)	28 (17.5)	42 (30.2)	
Proper muscle	62 (20.7)	40 (25.0)	22 (15.8)	
Subserosa	73 (24.4)	42 (26.2)	31 (22.3)	
Serosa/adjacent organ	94 (31.4)	50 (31.2)	44 (31.7)	
Lymph node metastasis	183 (61.4)	106 (66.7)	77 (55.4)	0.061
Lymphovascular invasion	198 (66.4)	120 (75.5)	78 (56.1)	< 0.001
Perineural invasion	116 (38.8)	64 (40.0)	52 (37.4)	0.734
Overall pathologic stage (AJCC 7th edition)				0.379
I	90 (30.1)	44 (27.5)	46 (33.1)	
II	96 (32.1)	50 (31.2)	46 (33.1)	
III	113 (37.8)	66 (41.2)	47 (33.8)	

EGC, early gastric cancer; AGC, advanced gastric cancer, WD, well-differentiated; MD, moderately differentiated; PD, poorly differentiated; SRC, signet ring cell; SRCa, signet ring cell carcinoma; and AJCC, American Joint Committee.

### The rate of claudin 18.2 positivity

**Figure 1:**
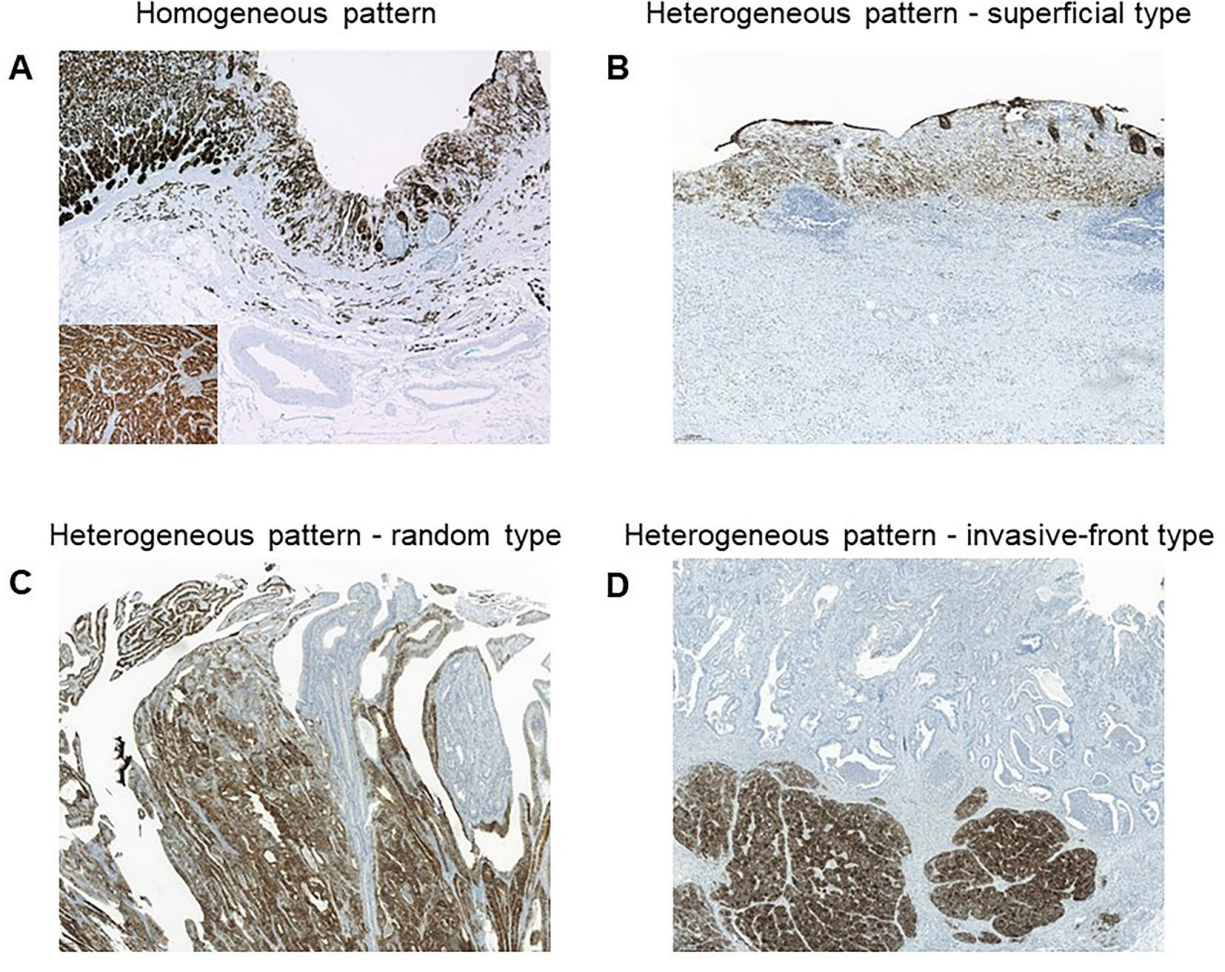
Homogeneous and heterogeneous expression patterns of claudin 18.2. (**A**) Homogeneous pattern, (**B**) Heterogeneous superficial pattern, (**C**) Heterogeneous random pattern, and (**D**) Heterogeneous invasive-front pattern.

Figure 1 shows some representative examples of claudin 18.2 overexpression by immunohistochemistry (IHC). In the overall study population with stage I–III tumors, claudin 18.2 overexpression was noted in 139 out of 299 patients (46.5%). Among these, a homogenous expression pattern was noted in 96 (69.0%), whereas 43 (31.0%) had a heterogeneous expression pattern, with most of them having a random expression pattern (n = 40). As shown in Fig. 1, the superficial and invasive front patterns have different expression intensities depending on the anatomic location of the tumor.

Claudin 18.2 overexpression was positive in 51.1%, 47.9%, and 41.6% of patients with stage I, stage II, and stage III disease, respectively.

### Clinicopathological characteristics according to claudin 18.2 expression

We next analyzed clinicopathological characteristics according to claudin 18.2 overexpression. Claudin 18.2 overexpression was associated with a younger age (median 61 vs. 66 years, P < 0.001) (Table 1). The proportion of male patients tended to be lower in the claudin 18.2-positive group (62.6% vs. 73.8%, P = 0.051). Claudin 18.2 positivity was more frequently observed in early gastric cancer (EGC) (30.2% vs. 17.5%, P = 0.014) (Table 1). Among patients with AGC, the proportion of Borrmann type 4 tumor was higher in the claudin 18.2-positive subgroup (20.6% vs. 10.6%, P = 0.008). Claudin 18.2-positive tumors had a higher proportion of diffuse histological type (48.2% and 33.1%, P = 0.011) (Table 1).

Claudin 18.2-positive tumors showed a trend toward a lower proportion of lymph node metastasis (55.4% vs. 66.7%, P = 0.061). Claudin 18.2-positive tumors were associated with a lower proportion of lymphovascular invasion (56.1% vs. 75.5%, P < 0.001), but there was no difference in the proportion of patients with perineural invasion (37.4% vs. 40.0%, P = 0.734) (Table 1).

The rate of claudin 18.2 positivity was significantly higher in HER2-negative tumors than in HER2-positive tumors (48.7% vs. 15.8%, P = 0.011) (Supplementary Table 1), whereas the claudin 18.2 positivity rate was higher in Epstein-Barr virus (EBV)-positive tumors (72.2% vs. 45.3%, P = 0.049) (Supplementary Table 2).

### Survival outcomes according to claudin 18.2 overexpression

**Figure 2:**
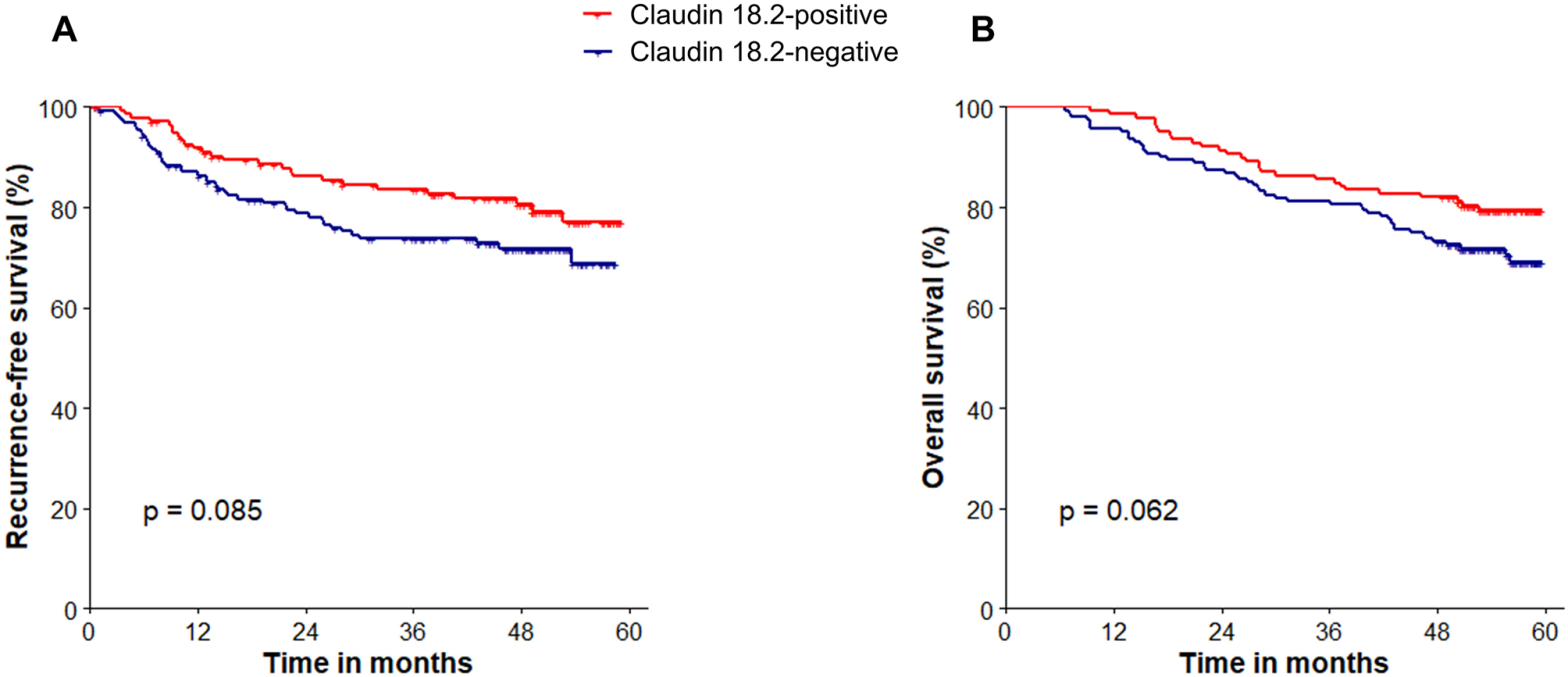
Survival outcomes according to claudin 18.2 positivity in the overall study population. (**A**) Recurrence-free survival and (**B**) Overall survival.

During a median follow-up period of 55.7 months (range 48.1–59.5 months), those with claudin 18.2-positive tumors tended to have favorable recurrence-free survival (RFS) (3-year RFS rate 83.6% and 73.7%, P = 0.085) and OS (3-year OS rate 85.6% vs. 81.3%, P = 0.062), respectively, as compared to those with claudin 18.2-negative tumors in the overall study population (Fig. 2).

**Figure 3:**
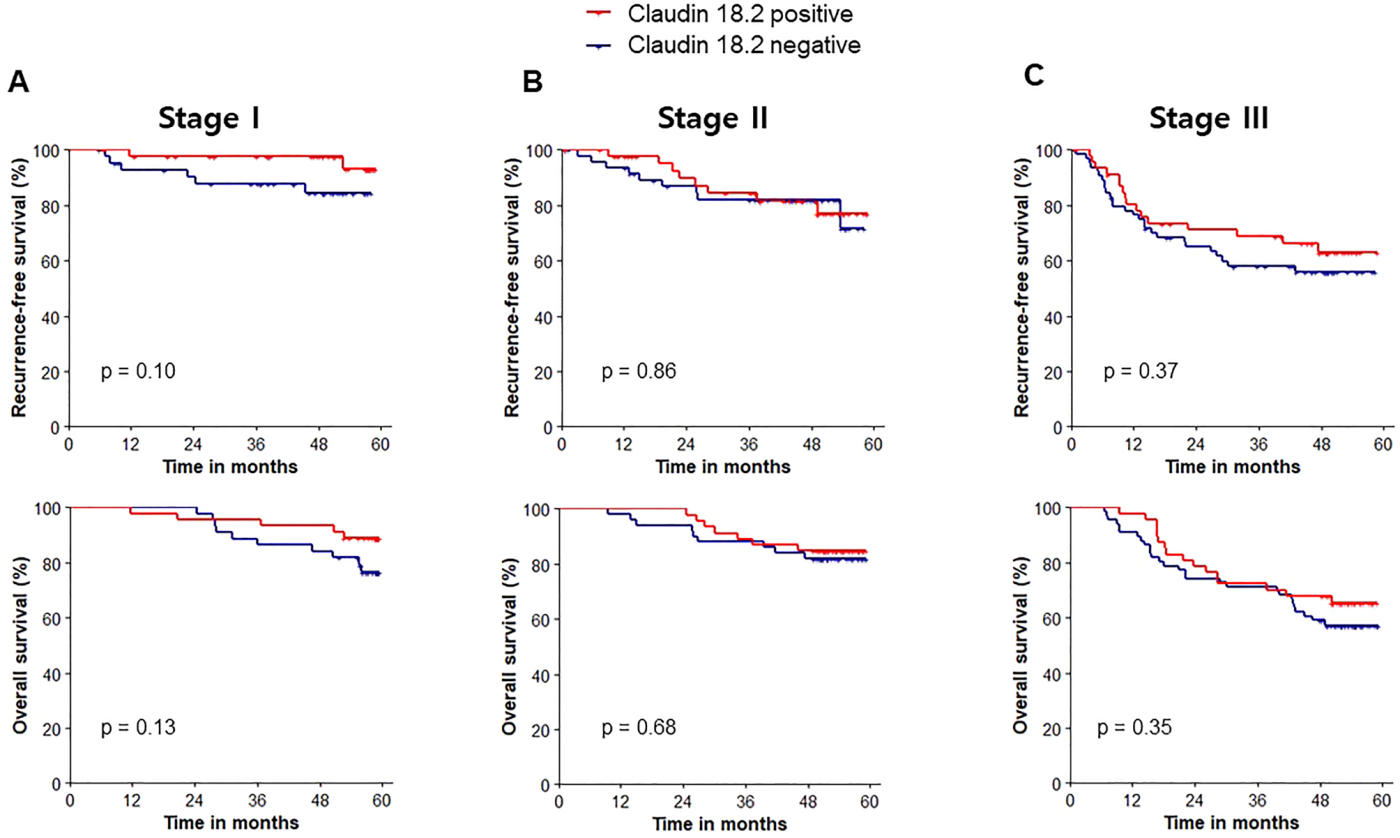
Survival outcomes according to claudin 18.2 positivity in each stage. Recurrence-free survival and overall survival for (**A**) Stage I, (**B**) Stage II, and (**C**) Stage III.

When survival outcomes were analyzed for each tumor stage, RFS was not different between claudin 18.2-positive and -negative tumors in each stage (P = 0.10, P = 0.86, and P = 0.37 for stage I, II, and III disease, respectively). Similarly, OS was comparable between the two groups (P = 0.13, P = 0.68, and P = 0.35 for stage I, II, and III disease, respectively) (Fig. 3).

In multivariate analysis, age > 60 years, perineural invasion, and stage were independent factors for RFS and OS, respectively (Table 2). Claudin 18.2-positivity was not an independent factor for RFS (hazard ratio [HR] 0.80, 95% confidence interval [CI] 0.48–1.32, P = 0.376) or OS (HR 0.77, 95% CI 0.47–1.25, P = 0.290).

**Table 2:** Factors associated with recurrence-free survival and overall survival.

Variables	Recurrence-free survival				Overall survival			
	Univariate analysis		Multivariate analysis		Univariate analysis		Multivariate analysis	
	HR (95% CI)	P value	HR (95% CI)	P value	HR (95% CI)	P value	HR (95% CI)	P value
Age > 60 years	1.38 (0.84–2.27)	0.197	1.75 (1.05–2.93)	0.032	1.65 (1.02–2.68)	0.043	1.97 (1.20–3.26)	0.008
Male sex	1.03 (0.61–1.72)	0.917	1.25 (0.73–2.15)	0.415	0.92 (0.57–1.49)	0.734	1.07 (0.65–1.76)	0.799
Lymphovascular invasion	1.63 (0.94–2.83)	0.081	0.90 (0.48–1.68)	0.745	1.75 (1.03–2.98)	0.039	1.00 (0.55–1.83)	0.993
Perineural invasion	4.20 (2.52–6.99)	< 0.001	3.35 (1.86–6.01)	< 0.001	2.92 (1.84–4.65)	< 0.001	2.74 (1.58–4.75)	< 0.001
Lauren (reference: intestinal)*			–	–			–	–
Diffuse subtype	0.96 (0.56–1.65)	0.890	–	–	0.85 (0.51–1.44)	0.551	–	–
Mixed + indeterminate subtype	0.97 (0.51–1.83)	0.919	–	–	1.03 (0.58–1.85)	0.911	–	–
Stage (reference: stage I)								
II	2.25 (0.97–5.22)	0.059	1.62 (0.65–4.06)	0.304	1.09 (0.54–2.21)	0.813	0.78 (0.37–1.68)	0.530
III	5.57 (2.61–11.87)	< 0.001	3.36 (1.36–8.35)	0.009	3.06 (1.70–5.53)	< 0.001	1.87 (0.90–3.87)	0.094
Claudin 18.2-positivity	0.65 (0.40–1.06)	0.087	0.80 (0.48–1.32)	0.376	0.64 (0.40–1.03)	0.064	0.77 (0.47–1.25)	0.290

*Lauren classification was not included in multivariate analysis.

## Discussion

In this retrospective analysis, we investigated the rate of claudin 18.2 positivity and its possible associations with the clinicopathological characteristics and survival outcomes of patients with claudin18.2-overexpressing localized resectable gastric cancer. The overall rate of claudin 18.2 overexpression was 46.5% in resectable gastric cancer, with a slightly higher positivity rate among stage I tumors (51.1%). Claudin 18.2-positive tumors had a higher probability of tumor invasion limited to the mucosa and submucosa. The rate of lymph node metastasis tended to be lower, and lymphovascular invasion was less frequent. On the other hand, claudin 18.2 expression was associated with Borrmann type 4, which is in line with a previous report in the metastatic setting^9^, and with the diffuse histological type, as in previous reports^10,13^. In the overall study population, claudin 18.2 positivity tended to be associated with favorable survival outcomes, possibly owing to its association with a low invasion depth and a low frequency of lymph node metastasis. However, the survival outcomes were similar regardless of claudin 18.2 positivity when analyzed separately by each tumor stage, and it was not an independent factor for survival outcomes in multivariate analysis as in the metastatic setting^9^. Since this study was conducted specifically for resectable tumors based on the use of the same antibody clone and cut-off for claudin 18.2 positivity as in the phase 3 SPOTLIGHT and GLOW trials, the results of the current study will serve as a unique resource for applying already established zolbetuximab-based treatments in a localized setting.

The survival benefits of adding zolbetuximab to chemotherapy seen in the phase 3 studies^7,16^ suggest the possibility of applying zolbetuximab in a localized setting. The rate of claudin 18.2 positivity in localized resectable gastric cancer (46.5%) was overall consistent with that reported in the phase 3 trials in a palliative setting (38.5% and 38.4% in the SPOTLIGHT and GLOW studies, respectively). The overall high positive rate of claudin 18.2 points to a potential wide applicability of claudin 18.2-directed treatment in the peri-operative setting. Therefore, prospective adjuvant or neoadjuvant trials can be considered in patients with localized resectable gastric cancer.

Given that the sensitivity for detecting claudin 18.2 expression could be different according to the claudin 18.2 IHC clones^11^, the use of different staining clones in previous studies^10,13,14,17^ may not readily be interpreted in the context of the claudin 18.2-directed treatments used in recent phase 3 studies^7,16^. Although previous studies have characterized the clinicopathological features of claudin 18.2 positivity in advanced gastric cancer or gastroesophageal junction cancer using the same IHC clone (43-14A) used in the phase 3 studies, the implications of claudin 18.2 positivity in a resectable setting could be limited, because those studies included only a small proportion of patients with stage I–II tumors (10.6%)^15^, used different cut-offs for claudin 18.2 positivity^11,12^, and did not separately analyze patients in curative and metastatic settings^11,15^. Therefore, our study could provide additional practical insights for applying claudin 18.2-directed treatments such as zolbetuximab to patients with localized gastric cancer.

Our study also showed that about 30% of cases with claudin 18.2 positivity showed a heterogeneous expression pattern, which is in line with previous reports (40–50% with heterogenous expression of claudin 18.2 in gastric cancer)^10,14^. This suggests that heterogenous claudin 18.2 expression could potentially lead to discrepancies of claudin 18.2 positivity between endoscopic biopsies and surgical specimens. This heterogeneous claudin 18.2 expression was more pronounced in cases classified as claudin 18.2 negative (less than 75% moderate-to-strong positivity in tumor cells, data not shown). In particular, the different expression patterns depending on the region of the tumor indicate that claudin 18.2 expression in endoscopic biopsies may not represent the whole tumor. The correlation between the degree of heterogeneous claudin 18.2 expression and its diagnostic and therapeutic implications in the context of claudin 18.2-directed treatments will need to be further explored in future studies.

While claudin 18.2 overexpression is not a prognostic factor, it serves as a predictive biomarker for claudin 18.2-directed therapies. In our analysis, claudin 18.2 overexpression was associated with a higher probability of tumor invasion limited to the mucosa and submucosa and a lower rate of lymphovascular invasion, but it was also associated with Borrmann type 4 and diffuse histological type. This suggests that there could be unknown factors related to claudin 18.2 that differentially contribute to a different tumor biology, prognosis, and therapeutic response of gastric cancers with claudin 18.2 overexpression. Therefore, for more precise stratification of claudin 18.2-overexpressing gastric cancer, efforts should be made to delineate additional (genetic) biomarkers such as *CLDN18-ARHGAP26/6* fusions, which are reportedly more prevalent in diffuse-type gastric cancers that involve lymphatic and distant metastases^18^.

There are some limitations to be considered in the current study. Its retrospective nature, the single center-based analysis, and the absence of a validation cohort may limit the interpretation and generalizability of our data. In addition, given that novel claudin 18.2-targeting agents other than zolbetuximab are currently under investigation^19–22^, the method of evaluating claudin 18.2 expression adopted in our analysis may not be universally applied to other claudin 18.2-targeted agents. Another limitation is that we did not evaluate the associations between claudin 18.2 expression and mismatch repair status and PD-L1, which should be explored in future studies of localized resectable gastric cancer.

In conclusion, claudin 18.2 was overexpressed in almost half of resectable gastric cancer patients. Claudin 18.2 overexpression was associated with a higher probability of tumor invasion limited to the mucosa and submucosa and a lower rate of lymphovascular invasion, but it was also associated with Borrmann type 4 and diffuse histological type in a localized setting. Claudin 18.2 overexpression was not an independent prognostic factor in a localized setting. Considering the substantial positive rate of claudin 18.2 in resectable gastric cancer and the survival benefits of zolbetuximab-based treatments in a metastatic setting, additional studies of claudin 18.2-directed perioperative treatments should be performed in the future.

## Patients and methods

### Study population

This retrospective analysis included 299 Korean patients with stage I–III resectable gastric cancer who underwent curative surgical resection (R0 resection) at Asan Medical Center (Seoul, South Korea) between March 2018 and February 2019. Clinical data regarding baseline patient characteristics, including age, sex, gross type, location, WHO classification, Lauren classification, depth of invasion, lymph node metastasis, lymphovascular invasion, and perineural invasion were retrospectively obtained by reviewing medical records. Disease stage and R0 resection rate were confirmed using the American Joint Committee (AJCC) cancer staging criteria 7th edition. For patients with stage I tumors, regular surveillance was conducted following surgical resection, whereas patients with stage II/III tumors were treated with adjuvant chemotherapy following D2 gastrectomy (S-1^23^ or capecitabine plus oxaliplatin^24^). This study was approved by the Institutional Review Board (IRB) of Asan Medical Center (IRB No. 2023–0154), and the requirement for informed consent from patients was waived because of the following de-identification process: after de-identifying the information of the research subjects, random research subject numbers were assigned. Data were analyzed based on the de-identified patient information, and all related documents, such as research data, were encrypted and stored in the researcher's private office so that only the researcher could access them, and the data were handled only by the researcher within that office.

This study was conducted in accordance with the ethical standards of the latest Declaration of Helsinki.

### Claudin 18.2 immunohistochemistry

All of the tissue specimens were representative sections obtained from surgical specimens. IHC was performed on 4 μm thick formalin-fixed paraffin-embedded sections, which were deparaffinized and re-hydrated using xylene and ethanol serially. Endogenous peroxidase was blocked by incubation in 3% H_2_O_2_ for 10 min, followed by heat-induced antigen retrieval. IHC labeling was performed using a claudin18.2 antibody (clone 43-14A, Ventana) with an autostainer (Benchmark XT, Ventana Medical Systems) and the OptiView DAB Detection Kit (Ventana Medical Systems), following the manufacturer’s protocol.

Considering the nature of claudin 18.2 as a surface protein, only membranous, linear staining was regarded to be positive. Any other immunoreactivity, such as granular expression in the cytoplasm or nucleus, was disregarded. The immunoreaction status was assessed using two well-established methods used in previous studies on claudin 18.2 expression in gastric cancer^7,16^. Claudin 18.2 overexpression was defined as moderate-to-strong positivity in at least 75% of the tumor cells. The intensity of expression was categorized into 4 tiers: absence of any expression (0), weak expression (+ 1), moderate expression (+ 2), or strong expression (+ 3).

Among patients with claudin 18.2 overexpression, the expression patterns of claudin 18.2 were classified based on the homogeneity and the location of expression in the tumor: (1) the homogeneous pattern was defined as expressed in more than 90% of the area with a moderate or strong intensity; (2) any heterogeneous pattern that did not fall into the category of the homogeneous pattern was further categorized into superficial, invasive front, and random patterns based on the previous literature reporting heterogenous expression patterns of claudin 18.2 and claudin 4^14,25^: the superficial pattern was defined by expression primarily in the mucosa, which showed an apparent decrease in immunostaining intensity toward the depths of the tumor^14^ ; the invasive front pattern was characterized by prominent expression in the deep invasive components of the tumor, with a decrease in the expression of the protein toward the tumor edge^25^; and the random pattern was defined as a pattern in which the distribution of expression was patchy with various intensities that were evenly distributed^14^.

HER2 IHC was performed using a Ventana anti-Her2/neu (4B5) rabbit monoclonal primary antibody (Ventana Medical System, Tucson, AZ), and HER2 positivity was determined using the gastric cancer consensus panel recommendations^26^.

The presence of EBV in the cancer cells was evaluated by EBV-encoded RNA, detected by chromogenic in situ hybridization, which was performed using a BenchMark XT autostainer (Ventana Medical Systems, Tucson, AZ) according to the manufacturer’s instructions.

### Statistical analysis

RFS was defined as the time from the date of surgical resection (index date) to disease recurrence or death, whichever came first. OS was defined as the time interval between the index date and the date of death from any cause. The Kaplan–Meier method was used to estimate survival outcomes, and the log-rank test was used for the comparison of survival outcomes among the subgroups. The chi-square test or Fisher's exact test was used to compare categorical variables among the subgroups. Cox proportional hazard modeling was used to assess the association between the examined factors and RFS and OS. In the multivariate analysis, variables with a potential relationship (P < 0.2) in the univariate analyses, along with age and sex, were included. As a result, 6 variables (i.e., age, sex, lymphovascular invasion, perineural invasion, stage, and claudin 18.2 positivity) were included in the multivariate Cox proportional hazard analysis, which was within the rule of ten (i.e., a minimum of 10 outcome events per predictor variable)^27,28^. A P-value of < 0.05 was considered statistically significant. Statistical analyses were performed using R (version 3.6.2; R Foundation for Statistical Computing, Vienna, Austria).

### Supplementary Information

Supplementary Information 1.

Supplementary Information 2.

Supplementary Information 3.

## Data Availability

Data can be made available to qualified investigators upon reasonable request to the corresponding authors.
