# Supplementary figures and images for: Clinicopathologic features and prognostic value of claudin 18.2 overexpression in patients with resectable gastric cancer
Source: Sci Rep. 2023 Nov 16;13:20047. doi: 10.1038/s41598-023-47178-6 (PMC10654731; doi:10.1038/s41598-023-47178-6)

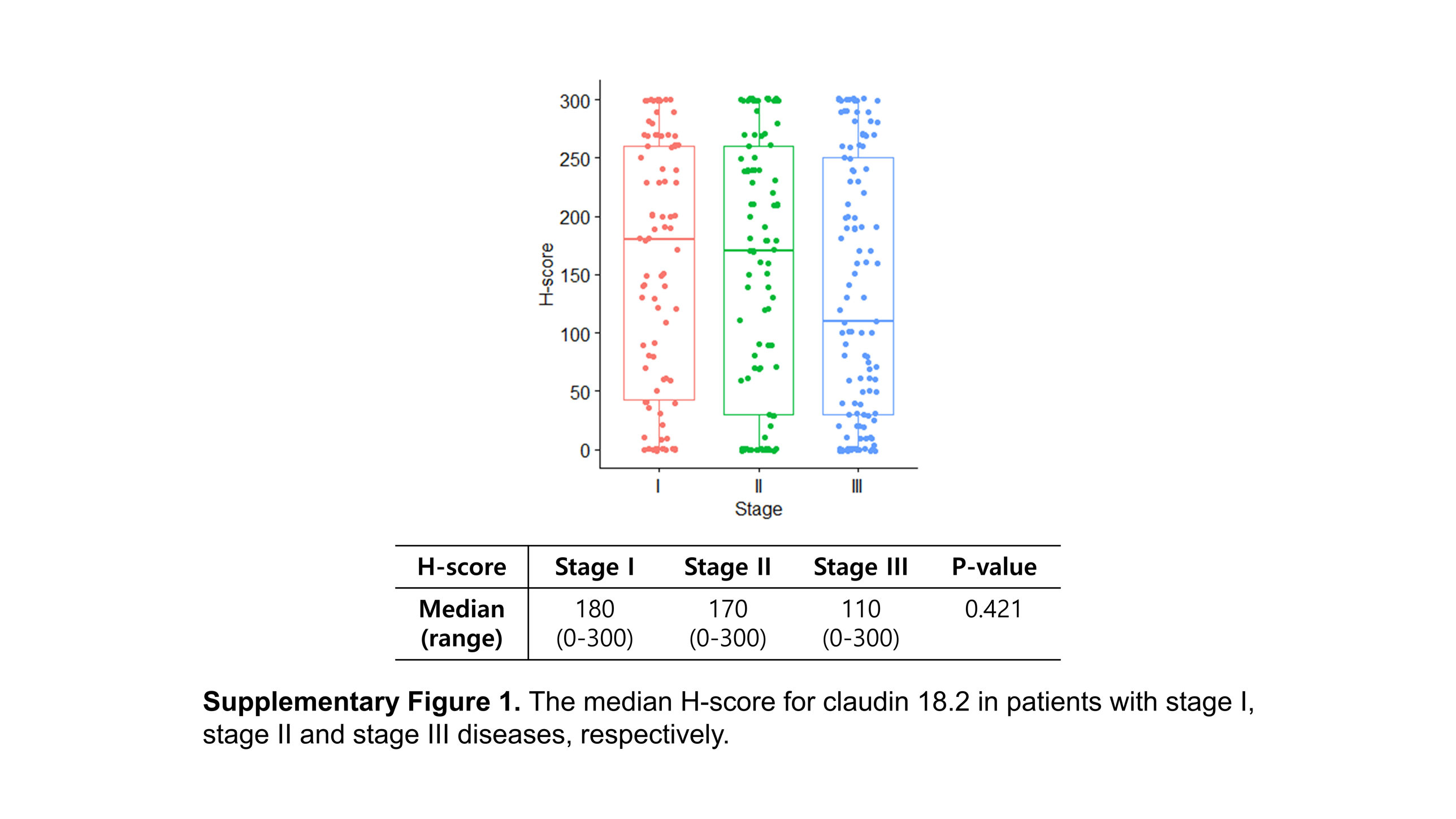

Supplement: Supplementary file 1 — Supplementary Information 1. [file 41598_2023_47178_MOESM1_ESM.jpg]
