# Supplementary material for: Clinicopathologic features and prognostic value of claudin 18.2 overexpression in patients with resectable gastric cancer
Source: Sci Rep. 2023 Nov 16;13:20047. doi: 10.1038/s41598-023-47178-6 (PMC10654731; doi:10.1038/s41598-023-47178-6)
Supplement: Supplementary file 2 — Supplementary Information 2. [file 41598_2023_47178_MOESM2_ESM.docx]

**Supplementary Table 1. Claudin 18.2 positivity according to EBV positivity**

|  | **EBV positive**  **(n = 18)** | **EBV negative**  **(n = 267)** | **p-value** |
| --- | --- | --- | --- |
| Claudin 18.2 negative | 5 (27.8) | 146 (54.7) | 0.049 |
| Claudin 18.2 positive | 13 (72.2) | 121 (45.3) |  |
