# Supplementary material for: Clinicopathologic features and prognostic value of claudin 18.2 overexpression in patients with resectable gastric cancer
Source: Sci Rep. 2023 Nov 16;13:20047. doi: 10.1038/s41598-023-47178-6 (PMC10654731; doi:10.1038/s41598-023-47178-6)
Supplement: Supplementary file 3 — Supplementary Information 3. [file 41598_2023_47178_MOESM3_ESM.docx]

**Supplementary Table 2. Claudin 18.2 positivity according to HER2 positivity**

|  | **HER2 positive**  **(n = 19)** | **HER2 negative**  **(n = 271)** | **p-value** |
| --- | --- | --- | --- |
| Claudin 18.2 negative | 16 (84.2) | 139 (51.3) | 0.011 |
| Claudin 18.2 positive | 3 (15.8) | 132 (48.7) |  |
